# Effect of Direct-Acting Antiviral Therapy on Thrombocytopenic Patients with Hepatitis C Virus-Related Chronic Liver Disease

**DOI:** 10.1155/2021/8811203

**Published:** 2021-05-23

**Authors:** Mahmoud Saif-Al-Islam, Usama M. Abdelaal, Mustafa Adel Younis, Hisham A. Alghany Algahlan, Safaa Khalaf

**Affiliations:** ^1^Tropical Medicine and Gastroenterology Department, Sohag University Hospital, Faculty of Medicine, Sohag, Egypt; ^2^Internal Medicine Department, Sohag University Hospital, Faculty of Medicine, Sohag, Egypt; ^3^Clinical and Chemical Pathology Department, Sohag University Hospital, Faculty of Medicine, Sohag, Egypt; ^4^Diagnostic Radiology Department, Sohag University Hospital, Faculty of Medicine, Sohag, Egypt

## Abstract

**Background and Aims:**

Thrombocytopenia is a common complication in patients with chronic hepatitis C virus (HCV) that increases the risk of bleeding. We aimed to analyze the hematologic effects of the new direct-acting antiviral (DAA) therapy, particularly on the platelet count in chronic HCV-infected patients with thrombocytopenia. *Patients and Methods*. One hundred thrombocytopenic patients chronically infected with HCV were included in a prospective study. All patients were eligible for receiving anti-HCV treatment with sofosbuvir-based regimens for 12 weeks, according to the protocol of the National Program for treatment of HCV in Egypt sponsored by the Ministry of Health.

**Results:**

At the end of treatment (EOT), there was a highly significant increase in platelet count (*p* < 0.001), a significant increase in white blood cells (WBCs) count (*p* ≤ 0.032), and a highly significant decrease in hemoglobin level (*p* < 0.001) as compared to pretreatment levels. Patients with mild to moderate hepatic fibrosis had significantly higher median and interquartile range (IQR) platelet count at baseline and EOT than those with advanced fibrosis and cirrhosis (*p* ≤ 0.023 and *p* < 0.001, respectively). There was more elevation in platelet count at EOT in patients with mild to moderate fibrosis than those with advanced fibrosis and cirrhosis. Out of the hundred patients, 73% showed improvement of platelet count, while 27% showed no improvement or even decrease in the platelet count.

**Conclusion:**

Sofosbuvir-based DAA therapy is a highly effective and safe treatment regimen that results in the improvement of platelet count in thrombocytopenic patients, particularly in mild to moderate stages of hepatic fibrosis.

## 1. Introduction

Chronic HCV infection affects up to 170 million people worldwide with approximately 25% of them develop cirrhosis [1, 2]. The continuous hepatic inflammation in HCV infection may cause hepatic fibrogenesis and eventually lead to cirrhosis [3]. Platelet count is strongly related to the degree of hepatic abnormalities especially in those patients with bridging fibrosis or cirrhosis [4, 5], and the change in platelet count correlated with the change in hepatic fibrosis following antiviral therapy [6, 7].

Thrombocytopenia in chronic HCV may result from either decreased platelet production by bone marrow or platelet sequestration within the spleen [8]. Also, intrahepatic accumulation of platelets with chronic hepatitis and cirrhosis may be one of the important contributory factors to thrombocytopenia [9]. The prevalence of thrombocytopenia in patients with chronic HCV ranged from 0.16% to 45.4%, however, more than half of the studies reported a thrombocytopenia prevalence of 24% or more. Such wide-range prevalence of thrombocytopenia is due to several factors; mainly the threshold used to define hematological abnormality and the severity of the underlying liver disease were not the same in all studies [10].

Early in the 2000s, dual therapy using a combination of pegylated interferon (Peg-IFN) and ribavirin was the standard treatment for HCV that required a duration of 48 weeks that could be extended to 72 weeks for partial responders [11]. However, treatment of chronic HCV has improved marvelously during the last 8 years. Newer DAA drugs directly inhibit the virus replication cycle and have the advantage of being oral treatment regimens with high antiviral efficiency and a favorable safety profile [12, 13]. Even in the case of advanced hepatic fibrosis, sustained virological response (SVR) was recorded over 90% with combination regimens of DAA therapy [14, 15].

Assessment of the hepatic fibrosis stage is important as the degree of hepatic fibrosis determines the need for antiviral treatment and may help to select the optimal duration of therapy as well as the most appropriate regimen [16]. Powerful antiviral agents have rapidly decreased the use of liver biopsy for detecting cirrhosis in patients with chronic viral hepatitis and have led to the development of noninvasive methods for evaluating fibrosis [17]. Liver stiffness measurement using ultrasound or magnetic resonance-based elastography techniques is one of the currently available noninvasive methods which have a great clinical value in the evaluation of liver fibrosis [18].

In this study, we aimed to analyze the hematologic effects of DAA therapy particularly on the platelet count in chronic HCV-infected patients with thrombocytopenia.

## 2. Patients and Methods

One hundred thrombocytopenic patients chronically infected with HCV were included in a prospective study. The study protocol was approved by our institute Medicine Ethical Committee, and written informed consents were obtained from all participants. The patients were eligible for receiving anti-HCV treatment with sofosbuvir-based regimens according to the protocol of the National Program for treatment of HCV in Egypt sponsored by the Ministry of Health. Chronic HCV infection was documented by positive anti-HCV antibody by enzyme-linked immunosorbent assay (ELISA) and detectable HCV RNA by polymerase chain reaction (PCR).

### 2.1. Inclusion Criteria

1. Patients with chronic HCV infection diagnosed by the presence of detectable serum anti-HCV antibodies by ELISA for more than 6 months and detectable HCV RNA by PCR
2. Patients with thrombocytopenia defined as a platelet count less than 150 × 10^3^/mL

### 2.2. Exclusion Criteria

1. Age below 18 years old
2. Patients with HCV and hepatitis B virus (HBV) coinfection
3. Patients with other causes of liver disease such as alcoholic liver disease and metabolic liver disease
4. Decompensated liver cirrhosis and/or hepatocellular carcinoma (HCC)

## 3. Methods

All patients were treated by sofosbuvir-based regimens for 12 weeks. Twenty-nine patients received dual therapy (sofosbuvir 400 mg once daily and daclatasvir 60 mg once daily) and seventy-one patients received triple therapy (sofosbuvir 400 mg once daily, daclatasvir 60 mg once daily and ribavirin 800 mg daily in patients who had single criterion, and 600 mg daily, in patients who had two or more criteria from the difficult to treat group).

According to the Modified National Program for the treatment of HCV in Egypt (provided by the National Committee for Control of Viral Hepatitis (NCCVH) in 2008) [19]:

(1) Easy to treat group: receiving sofosbuvir and daclatasvir
  - Treatment-naïve
  - Total bilirubin ≤ 1.2 mg/dl
  - Serum albumin ≥ 3.5 g/dl
  - INR ≤ 1.2
  - Platelet count ≥ 150 × 10^3^/ml
(2) Difficult to treat group: receiving sofosbuvir, daclatasvir, and ribavirin
  - Peg-IFN treatment-experienced
  - Total serum bilirubin ≥ 1.5 mg/dl
  - Serum albumin ≤ 3.2 g/dl
  - INR ≥ 1.2
  - Platelet count ≤ 100 × 10^3^/ml

### 3.1. Pretreatment Evaluation

- Complete history taking and thorough clinical examination
- Laboratory investigations

Ten milliliters of blood was drawn from each patient; 2 ml in a K2-EDTA vacutainer for complete blood count (CBC), 4 ml in lithium heparin vacutainer for liver function test, and 4 ml in gel vacutainer for serology and PCR. Vacutainers were (BD) (Becton Dickinson Systems, San Jose, CA, USA).

- CBC was carried out using the hematology analyzer Abbott Cell-Dyn Ruby (Abbott Laboratories, Abbott Park, IL, USA)
- *Liver Function Tests*. Including albumin, total bilirubin, alanine aminotransferase (ALT), and aspartate aminotransferase (AST) levels were carried out using an AU480 chemical analyzer (Beckman Coulter, Tokyo, Japan)
- *Serology*. HCV antibody was carried out using the Architect® i2000SR anti-HCV (CIA) chemiluminescence system (Abbott Laboratories, Diagnostics Division, Abbot Park, IL, USA)
- Quantitative HCV RNA level by Real-time PCR using QIAcube system (Qiagen, Hilden, Germany) with Spin tubes protocols (Qiagen) and The StepOne™ Real-Time PCR System (Applied Biosystems, Life Technologies, Foster City, CA, USA)

(III) *Abdominal Ultrasonography*. To evaluate the liver size, surface and parenchyma, portal vein (PV) diameter, spleen size, and the presence or absence of ascites or HCC
(IV) *Liver Stiffness Measurement*. Using shear wave elastography. The degree of hepatic fibrosis was classified using the METAVIR score with the following cutoffs: F0 (0.8 : 1.19 m/s), F1 (1.2 : 1.34 m/s), F2 (1.35 : 1.60 m/s), F3 (1.61 : 2 m/s), and F4 > 2 m/s (cutoff value for liver cirrhosis) [20]

### 3.2. Technique

We used a multihertz convex transducer (3-5 MHz); Toshiba Xario 200-platinum canon medical. Through the intercostal acoustic window with minimal rib shadowing optimizing visualization of the liver tissue, resting respiratory motion, upper right lobe scanning (segment 7 and 8) was done in an area free of lesion and vessels. The region of interest (ROI) was chosen about 1 cm from the liver capsule. Two circles are selected within homogeneous uniform color within the box, using the reading of standard deviation (SD) lesion 20% and tabulate the results. Shear wave elastography (SWE) measurement was expressed as shear wave velocity (m/s).

### 3.3. End of Treatment (EOT) Laboratory Follow-Up Investigation

- CBC
- Liver function tests
- Serum HCV RNA levels were assessed at EOT and 12 weeks after

### 3.4. Statistical Analysis

Data were analyzed using SPSS version 16. Quantitative data were represented as mean ± standard deviation, median, and range. The student's *t*-test was used when the data were normally distributed, and the Mann–Whitney test was used when the data was not normally distributed to compare two groups. Paired data were compared using the Wilcoxon rank test. Qualitative data were presented as number and percentage, and a comparison of data was done by Chi-square test and Fischer exact test when suitable. Pearson's and Spearman's correlation coefficient tests were used to evaluate the association between platelet count and quantitative and qualitative data, respectively. Multivariate logistic regression analysis was used to detect the predictors of nonimprovement in platelet count. Graphs were produced by SPSS program or Excel sheet. *p* value was considered significant if it was less than 0.05.

## 4. Results

**Figure 1:**
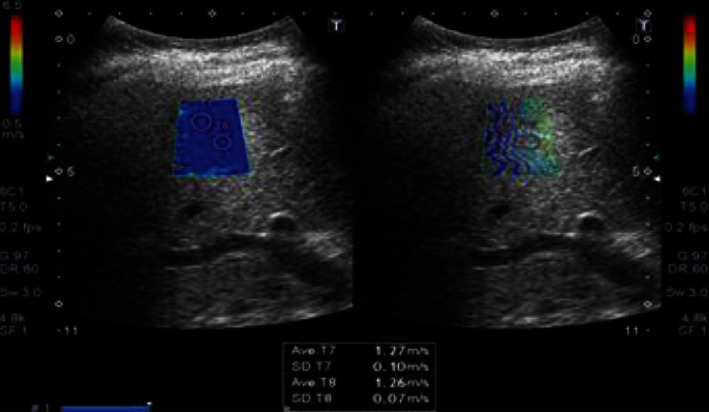
Diffuse hepatic parenchymal disease with F1 METAVIR score expressed as shear wave velocity (1.27 m/s).

**Figure 2:**
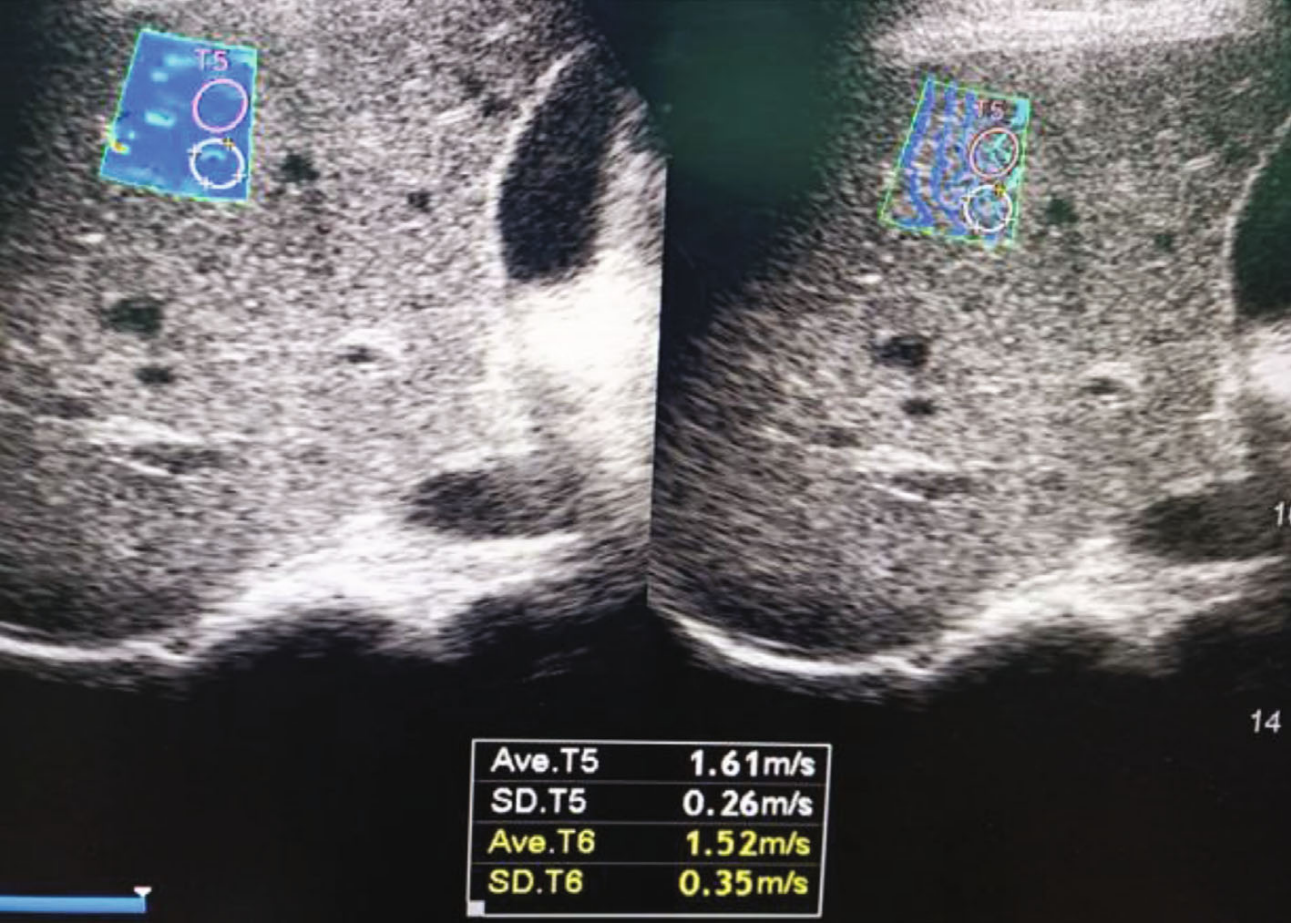
Diffuse hepatic parenchymal disease with F2 METAVIR score expressed as shear wave velocity (1.61 m/s).

**Figure 3:**
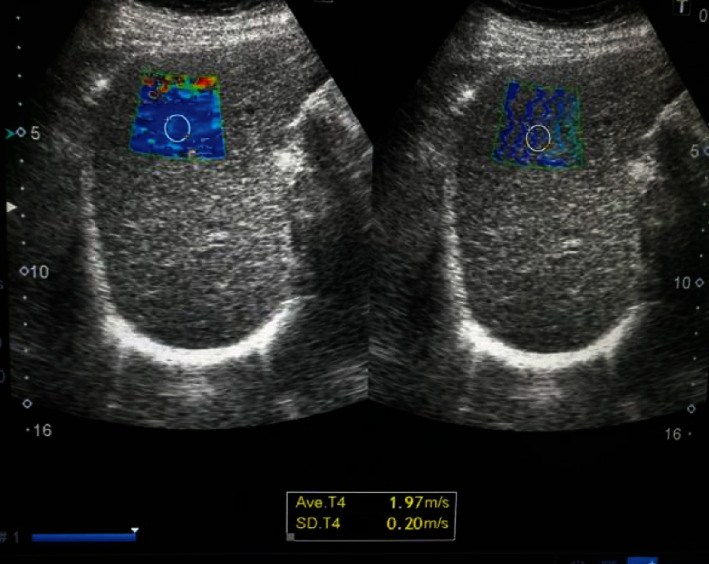
Diffuse hepatic parenchymal disease with F3 METAVIR score expressed as shear wave velocity (1.97 m/s).

**Figure 4:**
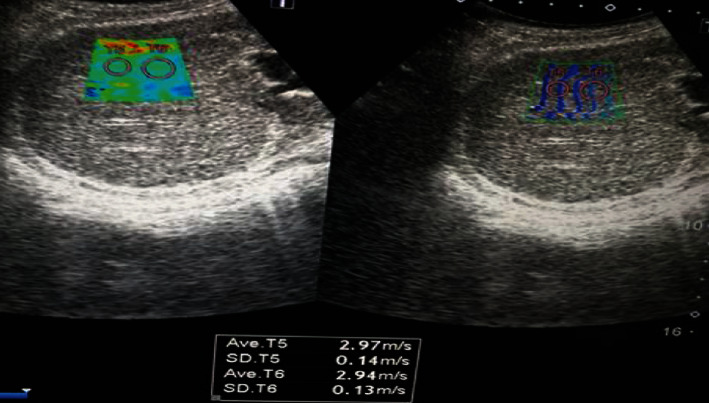
Diffuse hepatic parenchymal disease with F4 METAVIR score expressed as shear wave velocity (2.97 m/s).

A hundred patients were included in the study with a mean age of 56.6 ± 10.2 years, 56% were males and 44% were females, and 57% were known to have compensated liver cirrhosis. Liver stiffness measurements showed 23% of patients had mild to moderate hepatic fibrosis (F0, F1, and F2) (Figures 1 and 2) and 77% of patients had advanced hepatic fibrosis and cirrhosis (F3 and F4) (Figures 3 and 4). The baseline characteristics of the studied patients were shown in Table 1.

At the EOT, there was a highly significant increase in platelet count (*p* < 0.001), a significant increase in WBCs count (*p* ≤ 0.032), and a highly significant decrease in hemoglobin level (*p* < 0.001) as compared to pretreatment levels (Table 2).

**Figure 5:**
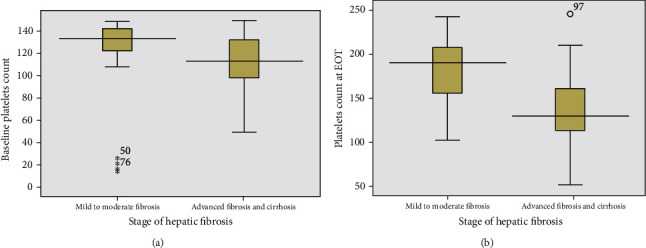
Platelet count at baseline (a) and end of treatment (b) in relation to different stages of hepatic fibrosis.

Patients with mild to moderate hepatic fibrosis (F1 and F2) had significantly higher median and IQR platelet count at baseline and EOT compared to those with advanced fibrosis and cirrhosis (F3 and F4) (*p* ≤ 0.023 and *p* < 0.001, respectively). There was more elevation in platelet count at EOT in patients with mild to moderate fibrosis than those with advanced fibrosis and cirrhosis (Table 3 and Figure 5).

There was a significant negative correlation between baseline platelet count and spleen size, stage of hepatic fibrosis, PCR, serum ALT, and AST (Table 4).

The changes in platelet count after DAA therapy were negatively correlated with baseline PV diameter, stage of hepatic fibrosis, and serum total bilirubin and positively correlated with baseline serum albumin. No correlation has been found between EOT platelet count and level of viremia or liver enzymes (Table 5).

Out of the hundred thrombocytopenic patients, 73% showed improvement of platelet count while 27% showed no improvement or even decrease in the platelet count. There were no significant differences in mean age, gender, or type of treatment between patients with and without improvement in platelet count. Most thrombocytopenic patients who had no improvement in platelet count had statistically significant higher stage of fibrosis, larger PV diameter, larger spleen size, higher baseline total bilirubin, and lower baseline albumin levels than those who had improvement in platelet count (*p* ≤ 0.006, *p* ≤ 0.009, *p* < 0.001, *p* ≤ 0.028, and *p* ≤ 0.013, respectively) (Table 6).

Multiple binary logistic regression analysis showed that raised levels of serum bilirubin were 5.93 times more significant than normal levels as a predictor of nonimprovement in platelet count on DAA therapy (*p* ≤ 0.033). The presence of splenomegaly was 1.55 times more significant than its absence as a predictor of nonimprovement in platelet count on DAA therapy (*p* < 0.001). The advanced stage of hepatic fibrosis and cirrhosis was 8.67 times more significant than mild to moderate stages of hepatic fibrosis as a predictor of nonimprovement in platelet count on DAA therapy (*p* ≤ 0.05) (Table 7).

## 5. Discussion

Treatment of chronic HCV is rapidly evolving. In 2014, the first NS5B RNA-polymerase inhibitor “sofosbuvir” was approved for the treatment of HCV. Since November 2015, sofosbuvir/daclatasvir with or without ribavirin became the main therapy in the National Program of Egypt [21]. Currently, DAA regimens are a milestone in the HCV eradication plan, with higher rates of SVR reaching 100% with certain DAA combinations [22]. Also, DAA therapy had a favorable safety profile compared with IFN-based treatment [23].

Thrombocytopenia in patients with chronic HCV is multifactorial; HCV promotes hepatic necroinflammation and fibrosis resulting in impaired liver function and decreased production and activity of thrombopoietin [8, 24]. Also, there are other mechanisms such as HCV-mediated bone marrow suppression and the presence of autoantibodies causing chronic immune thrombocytopenia [8, 25]. Karasu et al. demonstrated that in patients with chronic HBV and HCV, the stage of hepatic fibrosis is inversely correlated with platelet count [26]. Thus, changes in platelet count could represent a noninvasive tool to assess the evolution of the stage of liver disease and portal pressure [27].

In our study, we found that all the studied patients were responders at EOT and achieved SVR. Additionally, sofosbuvir-based regimens significantly improved platelet count at EOT compared to baseline. This was in agreement with Mohamed et al. who found a significant increase in platelet count in the majority of patients. However, their study did not select the thrombocytopenic patients [28]. Also, Hsu et al. found that there was a significant increase in the median platelet count from week 2 until EOT in all patients [6]. The precise mechanism underlying this improvement is unknown. However, this could be explained by the study of Pons et al. who reported an improvement in platelet count after DAA therapy with a negative correlation with splenic stiffness measurement and suggested that the increased platelet count is mainly due to decreased their splenic sequestration [7]. Also, in the era of IFN therapy, an international cohort study for many years demonstrated a linear increase in platelet count from the moment of SVR onwards associated with improvement in histopathological abnormalities of the liver, improvement in portal hypertension, and reversal of splenomegaly following antiviral therapy [4].

In the present study, we found that the baseline platelet count in patients with mild to moderate hepatic fibrosis was significantly higher than that in patients with advanced fibrosis and cirrhosis. This was in agreement with Osada et al. who observed significant differences in platelet count in different stages of liver disease and found a decrease in platelet count with the progression of hepatic fibrosis [29]. Kondo et al. reported that in patients with chronic hepatitis or cirrhosis, the accumulation of platelets in hepatic tissues increased along with an increase in histopathological damage, and blood platelet count significantly decreased in concordance with the severity of liver damage [9]. Our result supports this suggestion as to the more the fibrosis stage the more is the thrombocytopenia.

Also, we found that EOT patients with mild to moderate fibrosis had more elevation in platelet count than patients with advanced fibrosis and cirrhosis. This could reflect the more histopathological improvement in mild to moderate stages of fibrosis than advanced stages of fibrosis. Poynard et al. concluded that one of the factors independently associated with significant fibrosis reduction after HCV antiviral therapy was the baseline stage of hepatic fibrosis [30]. Petrenkienė et al. found that anti-HCV therapy decreased liver necroinflammation; however, advanced fibrosis has not been influenced by the 24-week treatment success [31]. Several studies reported that improvement in platelet count was correlated with the regression of hepatic fibrosis following SVR12 [32, 33]. This could explain why advanced stages of hepatic fibrosis were associated with poor improvement of platelet count in our study.

Several previous studies have shown that the degree of thrombocytopenia correlates with the extent of chronic hepatic injury, and platelet count is one of the factors that reflect the degree of hepatic fibrosis and the severity of liver cirrhosis [4, 34].

In our study, we found that platelet count negatively correlated with the presence of splenomegaly, stage of hepatic fibrosis, level of viremia, and elevated liver enzyme. Osada et al. reported that splenomegaly and impaired liver function were correlated well with thrombocytopenia [29]. Several previous studies have shown an inverse correlation between splenic size and platelet count in chronic HCV-infected patients [35, 36]. Also, platelet count has been inversely correlated with the severity of hepatic fibrosis [37, 38]. A significant correlation was found between the degree of thrombocytopenia and the level of viral load [39, 40]. There was a significant reversed relationship between platelet count and serum ALT levels with higher ALT levels in patients with low platelet count [40].

In the current study, the changes in platelet count after DAA therapy were negatively correlated with baseline PV diameter, stage of hepatic fibrosis, and serum total bilirubin and positively correlated with baseline serum albumin. A limitation of our study, however, is that imaging data were available only at baseline time. Thus, we could not investigate the correlation between the change in platelet count and change in spleen size or fibrosis stage as Van der Meer and his colleagues who found that the reduction of the spleen size and regression of liver fibrosis are positively associated with the increase in platelet counts among patients with SVR [4].

In our study, the majority of patients (*n* = 73) showed improvement in platelet count while only 27 patients showed no change or even decrease in the platelet count. Most thrombocytopenic patients who had no improvement in platelet count had a significantly higher stage of fibrosis, more PV diameter, spleen size, and more impaired liver function (low serum albumin and elevated total bilirubin) compared to patients who had improvement in platelet count. But based on multivariate logistic regression analysis, the independent predictors of nonimprovement in platelet count were raised serum total bilirubin, presence of splenomegaly, and advanced stage of hepatic fibrosis and cirrhosis.

In our study, there was a significant increase in WBCs count from 5.88 ± 2.24 to 6.38 ± 2.48. Our results suggest that successful HCV eradication leads to improvement in hepatic necroinflammatory activity reflected in the normalization of the liver enzymes. As there is no longer liver injury, the process of accumulation of platelets and recruitment of leukocytes to the injured hepatic tissue is terminated reflected in an increase in platelet count and WBC count in the peripheral blood. In chronic viral hepatitis, platelets interact with the hepatic sinusoidal endothelium while circulating in the injured liver and recruit effector cells and proteins. This activity causes a self-perpetuating cycle of platelet and leukocyte accumulation, resulting in hepatocellular injury [41, 42]. Also, we found a statically significant mild decrease in hemoglobin levels at EOT as compared to pretreatment levels. This was in agreement with the known side effects of ribavirin. Also, Cho et al. found a significant decrease in hemoglobin from 13.8 ± 2.4 g/dl to 12.2 ± 2 g/dl (*p* = 0.044) following sofosbuvir-based therapy. However, no patient needed a blood transfusion [43].

## 6. Conclusion

Sofosbuvir-based DAA therapy is a highly effective and safe treatment regimen that results in the improvement of platelet count in thrombocytopenic patients particularly in mild to moderate fibrosis stages.

## Figures and Tables

**Table 1:** Baseline characteristics of the studied patients.

Variables	(Mean ± SD, *n* %)
Age (years)	56.6 ± 10.2
Sex: *n* (%)	
Male	56 (56%)
Female	44 (44%)
Presence of cirrhosis: *n* (%)	
(i) Noncirrhotic	43 (43%)
(ii) Cirrhotic:	57 (57%)
Child A	50 (88%)
Child B	7 (12%)
Portal vein diameter (mm)	11.35 ± 2.05
Spleen size (cm)	14.18 ± 2.80
Stage of hepatic fibrosis: *n* (%)	
F0	6
F1	5
F2	12
F3	6
F4	71
Mild to moderate fibrosis (F0, F1, and F2)	23 (23%)
Advanced fibrosis and cirrhosis (F3 and F4)	77 (77%)
PCR (IU/ml)	1.8 × 10^6^ ± 2131108
Platelet count (×10^3^/mL)	112.55 ± 30.19
WBCs (×10^3^/mL)	5.88 ± 2.24
Hemoglobin (g/dL)	13.94 ± 1.65
Total bilirubin (mg/dL)	0.65 ± 0.33
Albumin (g/dL)	3.64 ± 0.56
ALT(IU/L)	71.24 ± 45.56
AST (IU/L)	84.29 ± 58.31
Treatment type: *n* (%)	
Dual	29 (29%)
Triple	71 (71%)

SD: standard deviation; *n*: number; PCR: polymerase chain reaction; WBCs: white blood cells; ALT: alanine aminotransferase; AST: aspartate aminotransferase.

**Table 2:** Hematological changes following antiviral therapy.

Variable	Baseline	End of treatment	Change	*p* value
Platelet count				
Mean ± SD	112.55 ± 30.19	146.91 ± 46.02	34.36 ± 48.17	≤0.001
Median (IQR)	119 (100.30 : 134)	145 (116.50 : 186)	26 (16.20 : 52)	
WBCs				
Mean ± SD	5.88 ± 2.24	6.38 ± 2.48	0.49 ± 2.27	≤0.032
Median (IQR)	5.4 (4.3 : 6.55)	5.75 (4.5 : 7.7)	0.35 (0.2 : 1.15)	
Hemoglobin				
Mean ± SD	13.94 ± 1.65	12.75 ± 1.51	−1.19 ± 0.14	≤0.001
Median (IQR)	13.95 (12.85 : 15.25)	12.60 (11.70 : 13.75)	-1.35 (1.15 : 1.5)	

SD: standard deviation; IQR: interquartile range; WBCs: white blood cells.

**Table 3:** Platelet count in relation to different stages of hepatic fibrosis.

	Mild to moderate hepatic fibrosis (*N* = 23)	Advanced hepatic fibrosis and cirrhosis (*N* = 77)	*p* value
Baseline platelet count (×10^3^)			
Mean ± SD	114.09 ± 45.99	112.09 ± 23.96	≤0.023
Median (IQR)	133 (122 : 142)	113 (98 : 133)	
12-week platelet count (×10^3^)			
Mean ± SD	185 ± 48	135 ± 39	≤0.001
Median (IQR)	190 (156 : 206)	129 (113 : 161)	

**Table 4:** Correlation between baseline platelet count, imaging, and laboratory data.

		Spleen size	PV diameter	Fibrosis stage	PCR	Total bilirubin	Serum albumin	ALT	AST
Platelet count	*p* value	≤0.047	≤0.372	≤0.022	≤0.01	≤0.601	≤0.173	≤0.03	≤0.045
	Correlation	-0.199	-0.090	-0.229	-0.256	-0.053	0.137	-0.217	-0.201

PV: portal vein; PCR: polymerase chain reaction; ALT: alanine aminotransferase; AST: aspartate aminotransferase.

**Table 5:** Correlation between EOT platelet count and baseline imaging and laboratory data.

		PV diameter	Spleen size	Fibrosis stage	PCR	Total bilirubin	Serum albumin	ALT	AST
Platelet count	*p* value	≤0.044	≤0.732	≤0.001	≤0.923	≤0.05	≤0.002	**≤**0.231	**≤**0.095
	Correlation	-0.0243	-0.095	-0.471	-0.056	-0.193	0.299	-0.127	-0.169

PV: portal vein; PCR: polymerase chain reaction; ALT: alanine aminotransferase; AST: aspartate aminotransferase.

**Table 6:** Patients with and without improvement of platelet count at EOT.

Variables	Patients with improvement in platelet count (*N* = 73)	Patients without improvement in platelet count (*N* = 27)	*p* value
Age (year)	55.99 ± 11.45	58.26 ± 5.54	**≤**0.225
Male/female (*n*, %)	39 (53%)/34 (47%)	17 (63%)/10 (37%)	**≤**0.394
Stage of hepatic fibrosis: (*n*, %)			
Mild to moderate fibrosis (F0, F1, and F2)	22 (30%)	1 (4%)	≤0.006
Advanced fibrosis and cirrhosis (F3 and F4)	51 (70%)	26 (96%)	
PV diameter (mm)	11.02 ± 1.98	12.24 ± 2	≤0.009
Spleen size (cm)	13.44 ± 2.51	16.17 ± 2.62	≤0.001
Baseline total bilirubin (mg/dL)	0.70 ± 0.35	0.89 ± 0.38	≤0.028
Baseline albumin (g/dL)	3.77 ± 0.55	3.50 ± 0.51	≤0.013
Baseline ALT (IU/L)	71.07 ± 40.40	71.70 ± 58.16	**≤**0.284
Baseline AST (IU/L)	82.20 ± 49.04	89.92 ± 79.01	**≤**0.666
Type of treatment: (*n*, %)			
Dual	24 (33%)	5 (18.5)	**≤**0.160
Triple	49 (67%)	22 (81.5%)	

PV: portal vein; ALT: alanine aminotransferase; AST: aspartate aminotransferase.

**Table 7:** Multivariate analysis of risk factors that hinder improvement in platelet count.

	Comparison	Odds ratio (95%C.I)	*p* value
Baseline total bilirubin (mg/dl)	Per increase in the level	5.93 (1.15-30.50)	≤0.033
Baseline albumin (g/dl)	Per decrease in the level	0.50 (0.17-1.48)	≤0.213
Spleen size (cm)	Per cm increase	1.55 (1.21-1.98)	≤0.001
Portal vein diameter (mm)	Per mm increase	1.1 (0.82-1.98)	≤0.523
Stages of fibrosis:			
(i) Mild to moderate	Per increase in stage	8.67 (0.96-77.96)	≤0.05
(ii) Advanced fibrosis and cirrhosis			

## Data Availability

All the authors have the full access to all of the data in the study, and they are fully responsible for the integrity of the data and the accuracy of the data analysis.

## Additional Points

*Place of Study*. Tropical Medicine and Gastroenterology Department, Sohag University Hospital, Sohag, Egypt. The study protocol was approved by our institute Medicine Ethical Committee (Date: 2019/2020; No. 1).
